# Supplementary material for: Pulmonary Inhalation of Biotherapeutics: A Systematic Approach to Understanding the Effects of Atomisation Gas Flow Rate on Particle Physiochemical Properties and Retained Bioactivity
Source: Pharmaceutics. 2024 Aug 1;16(8):1020. doi: 10.3390/pharmaceutics16081020 (PMC11359500; doi:10.3390/pharmaceutics16081020)
Supplement: Supplementary file 1 [file pharmaceutics-16-01020-s001.zip › pharmaceutics-3111516-supplementary.pdf]

Supplementary Materials

Table of Contents

Methods

- Spray Drying Preparation

Results

- Particle Size Distribution
- Bioactivity Retention
- Powder X-Ray Diffraction (PXRD)
- Shear Cell Test

Methods

Spray Drying Preparation:

Liquid feed stock solutions used for these tests were prepared using ultra-pure type II Millipore water. A 5% w/v of lysozyme was used for all spray drying runs. Trehalose was added additionally to the 5% w/v of lysozyme at varying excipient contents 1:1, 1:2 and 2:1 w/w. Samples were stirred for 1hr before processing. The Buchi B-290 mini spray dryer was used in open loop mode. This was used in conjunction with a dehumidifier set at -4 ˚C. A 2-fluid nozzle with a 0.7 mm nozzle tip diameter was used throughout the study.

Three atomisation gas flow rates were investigated: 473 L/h, 601 L/h and 742 L/h with a feed rate of 1.5 ml/min and the aspirator set to 100% (35.0 m3/h or 35000 L/h). All operating parameters are outlined in Table 1. The process was first stabilised using the solvent and once stable the feed stock was then pumped to the drying chamber. All samples were collected after 20-25 minutes of spray drying.

Results and Discussion

Particle Size Distribution

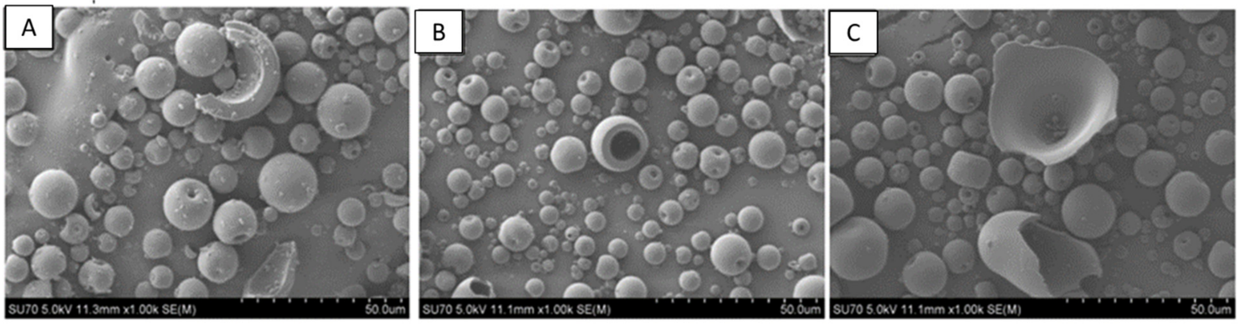

Figure S1. SEM images of particle thickness of (**A**) sample 1, (**B**) sample 2 and (**C**) sample 3 seen in Table 1.

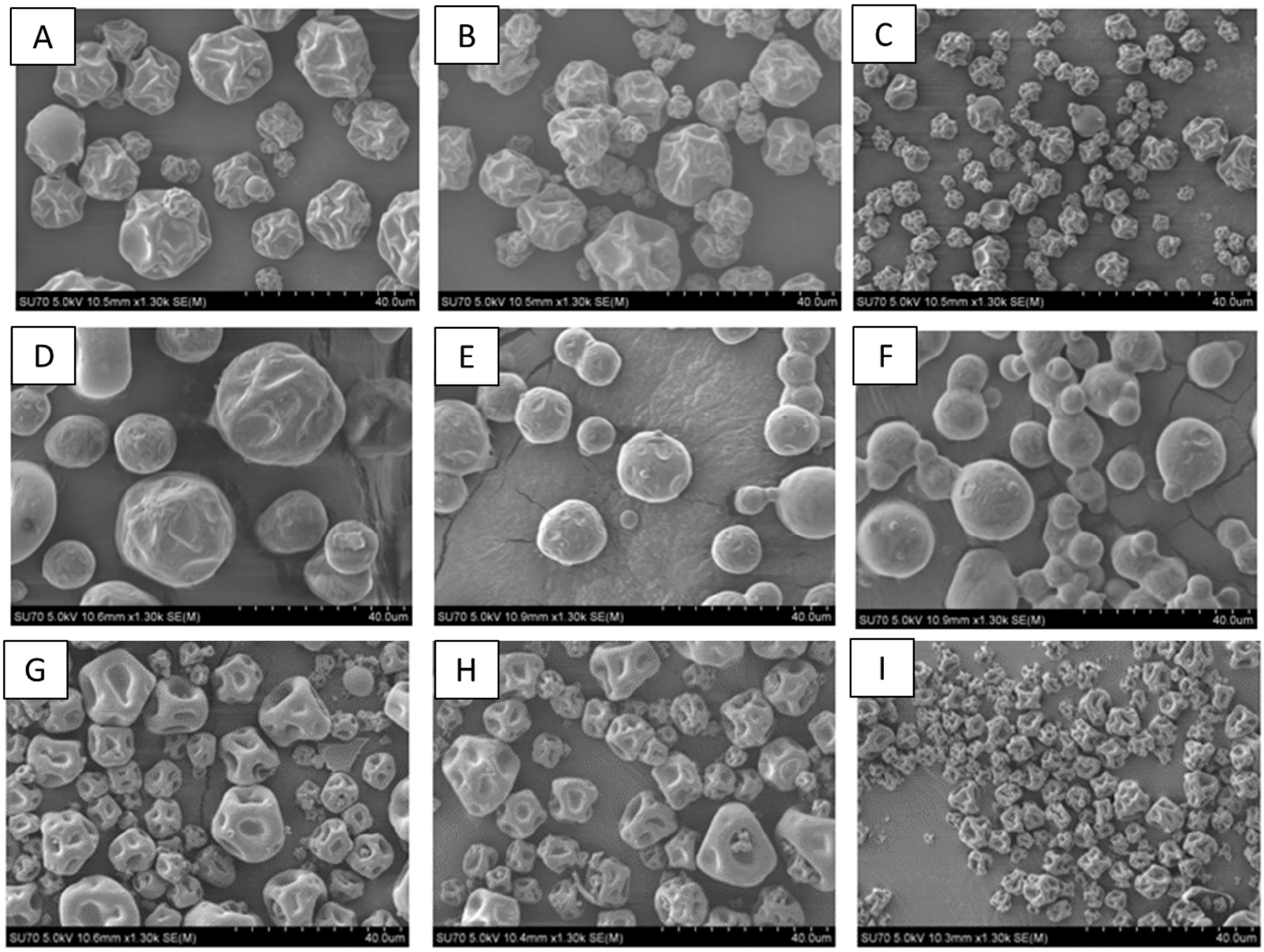

Figure S2. SEM images captured of spray dried lysozyme: trehalose samples, (**A**–**C**) are Samples 4, 7 and 10 spray dried at 1:1 ratio at atomising gas flow rate 473L/h, 601L/h and 742L/h respectively, (**D**–**F**) are Samples 6, 9 and 12 spray dried at 1:2 ratio at atomising gas flow rate 473L/h, 601L/h and 742L/h respectively, (**G**–**I**) are Samples 13, 14 and 15 spray dried at 2:1 ratio with elevated temperatures at atomising gas flow rate 473L/h, 601L/h and 742L/h respectively.

Table S1. Outlines the D_{10}, D_{50} and D_{90} for all spray dried samples.

ID	D_{10}	D_{50}	D_{90}
1	7.5	12.2	16.5
2	6.5	9.6	14.7
3	4.4	8.9	16.1
4	9.3	18.4	30.4
5	4.9	8.2	11.9
6	9.9	16.6	27.9
7	3.7	9.2	15.7
8	5.3	10.2	16.5
9	8.1	13.8	26.5
10	3.4	5.2	8.1
11	4.3	10.9	20.5
12	8.7	26.1	52.9
13	2.4	4.9	9.8
14	1.9	4.5	9.9
15	1.6	4.1	6.6

Bioactivity Retention

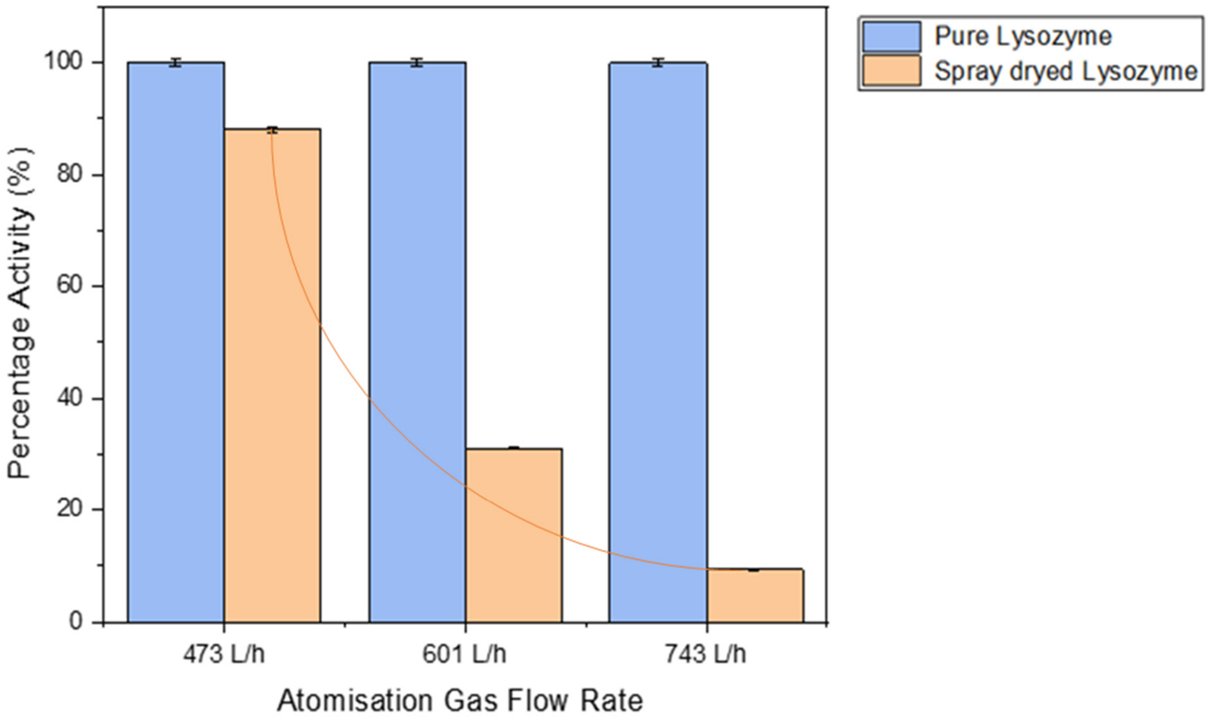

Figure S3. Comparative graph of the retained bioactivity before and after spray drying (Orange bars represent samples 1–3 in Table 1). A significant decrease in bioactivity retention is seen over the three atomisation gas flow rates.

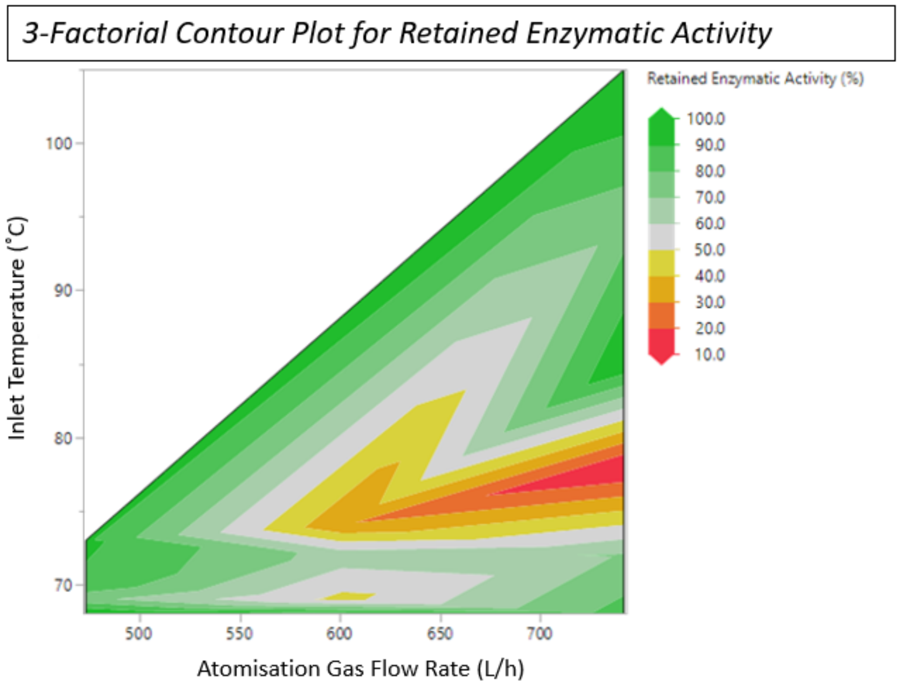

Figure S4. 3-Factorial contour plot for retained bioactivity of the effect of atomisation gas flow rate and inlet temperature.

Powder X-Ray Diffraction (PXRD)

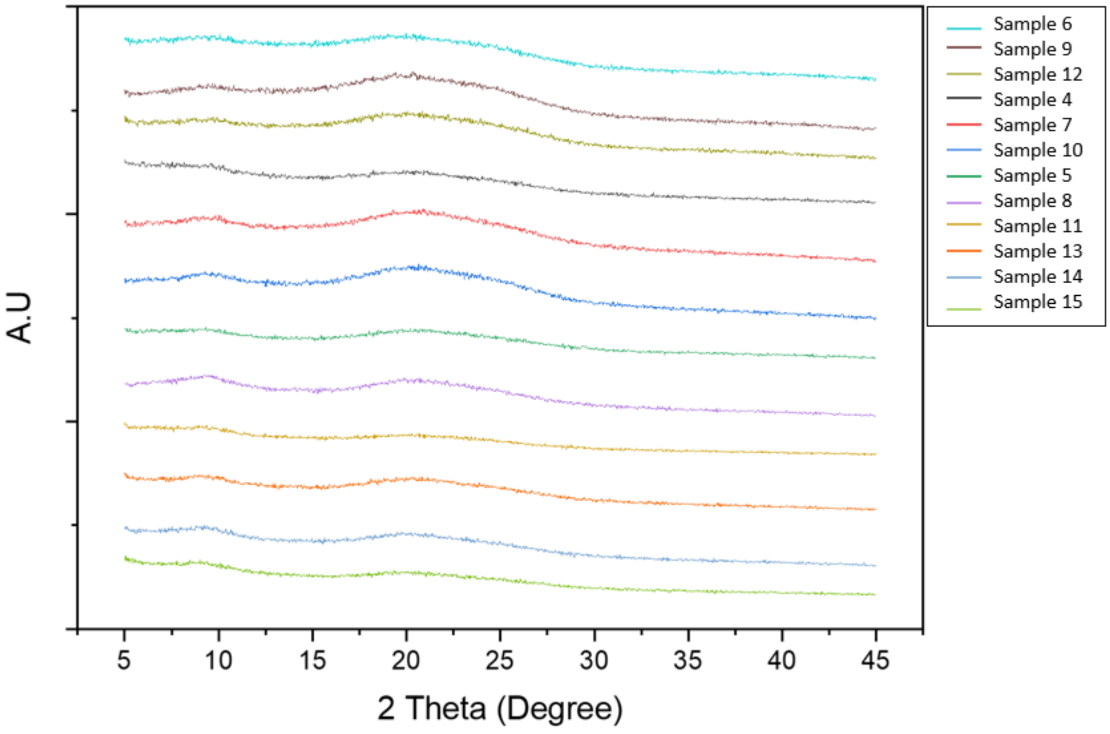

Figure S5. PXRD of spray dried lysozyme: trehalose at varying ratios (Table 1). No crystallinity could be detected in samples.

Shear Cell Test

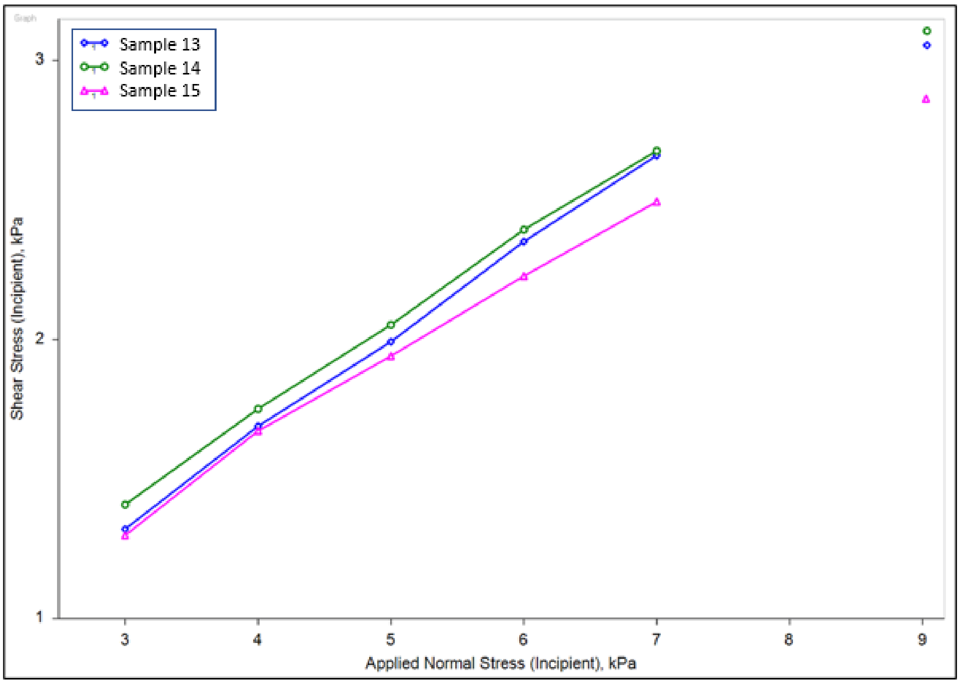

Figure S6. Shear test yield loci following the preshear at 9kPa normal stress for the optimised samples 13–15, spray dried at higher temperatures.
